# Supplementary material for: A robust targeted sequencing approach for low input and variable quality DNA from clinical samples
Source: NPJ Genom Med. 2018 Jan 15;3:2. doi: 10.1038/s41525-017-0041-4 (PMC5768874; doi:10.1038/s41525-017-0041-4)
Supplement: Supplementary file 1 — Supplemental Material [file 41525_2017_41_MOESM1_ESM.pdf]

SUPPLEMENTARY MATERIALS

A Robust Targeted Sequencing Approach for Low Input and Variable Quality DNA from Clinical Samples

Austin P. So[1,*], Anna Vilborg[1,*,§], Yosr Bouhlal[1], Ryan T. Koehler[1], Susan M. Grimes[2], Yannick Pouliot[1], Daniel Mendoza[1], Janet Ziegle[1], Jason Stein[1], Federico Goodsaid[1], Michael Y. Lucero[1], Francisco M. De La Vega[1,3], Hanlee P. Ji.[2,4,§]

[1]TOMA Biosciences, Foster City, CA, USA

[2]Stanford Genome Technology Center, Palo Alto CA, USA

[3]Department of Biomedical Data Science, Stanford University School of Medicine, Stanford CA, 94305

[4]Division of Oncology, and Stanford University School of Medicine, Stanford CA, USA

*These authors contributed equally to this work.

[§]Corresponding authors

Correspondences:
Anna Vilborg
Email: anna@tomabio.com

Hanlee P. Ji
Email: genomics_ji@stanford.edu

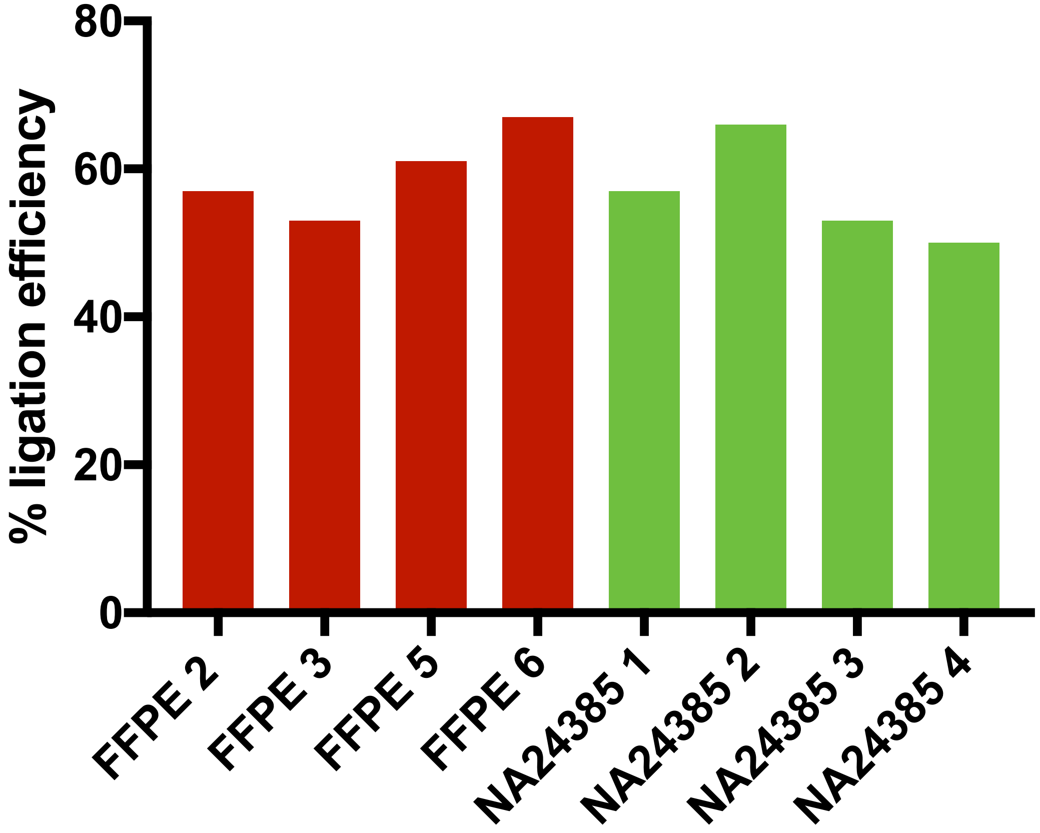

Supplementary Figure 1. Ligation efficiency in FFPE and reference samples

Four FFPE samples (FFPE 2, 3, 5 and 6 as listed in Supplementary table 2) and 4 NA24385 reference DNA samples were processed through the repair and kinasing steps of the TOMA 130-gene protocol and assayed for ligation efficiency by ddPCR (see Methods).

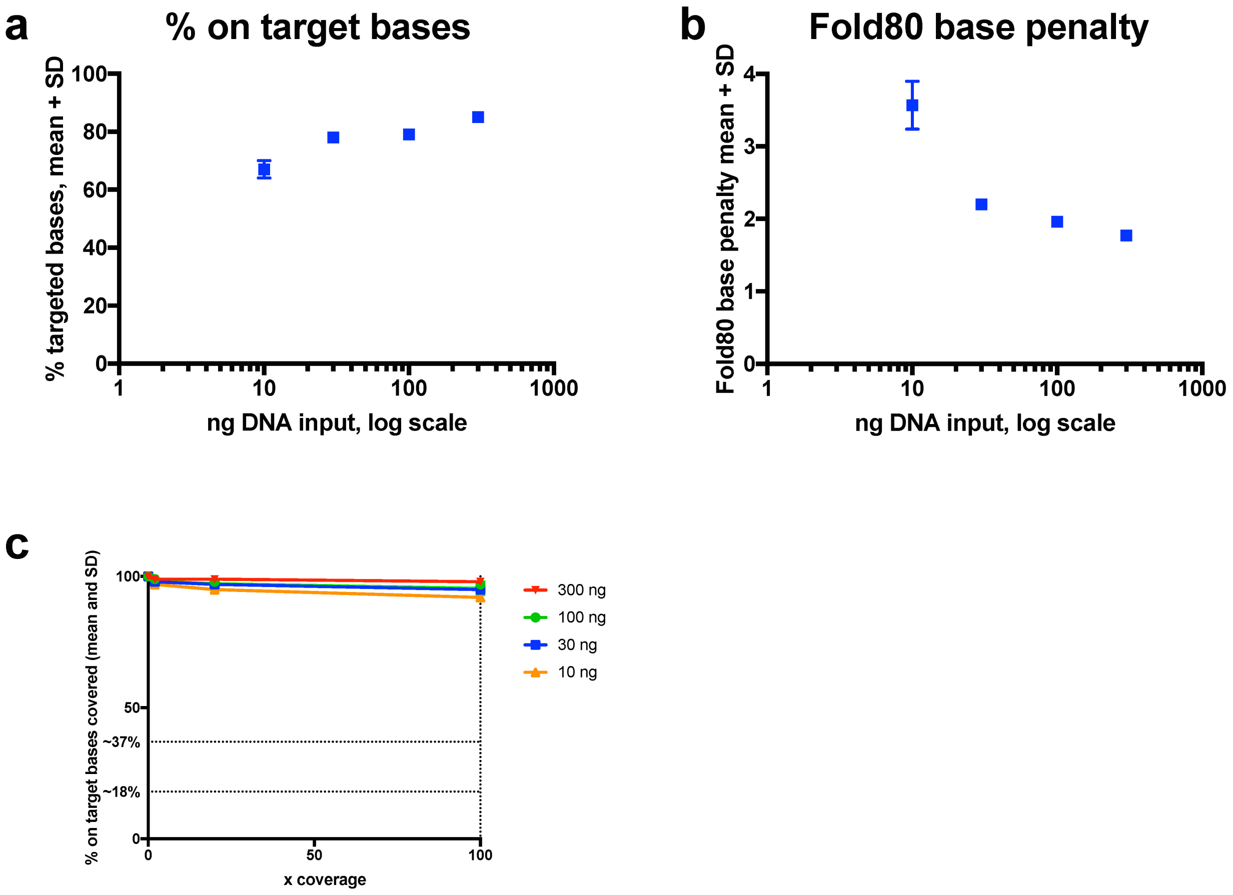

Supplementary Figure 2. Metrics from NA12878 mass titration experiment.

The percentage of the on-target bases (a) and the fold 80 base penalty (b) are shown as a function of DNA input. All data points show mean (squares) and **SD** (error bars), but the SD is too small to be visible for data points other than at 10 ng. (c) The percentage of targeted bases covered at 0X, 2X, 20X, and 100X as a function of DNA input (300 ng: red triangles, 100 ng: green circles, 30 ng: blue squares, 10 ng: yellow inverted triangles). All data points show mean (symbols) and SD (error bars) across four technical replicates, however the SD is smaller than the size of the symbol and therefore not visible). The dotted lines at ~18% and ~37% show the approximate range of % bases (lowest to highest) covered at 100X in whole exome sequencing of NA18943 using four separate whole exome protocols (Shigezmu et al, Science Reports 2015).

Shigemizu, D. et al. Performance comparison of four commercial human whole-exome capture platforms. Science Reports 5, 12742 (2015).

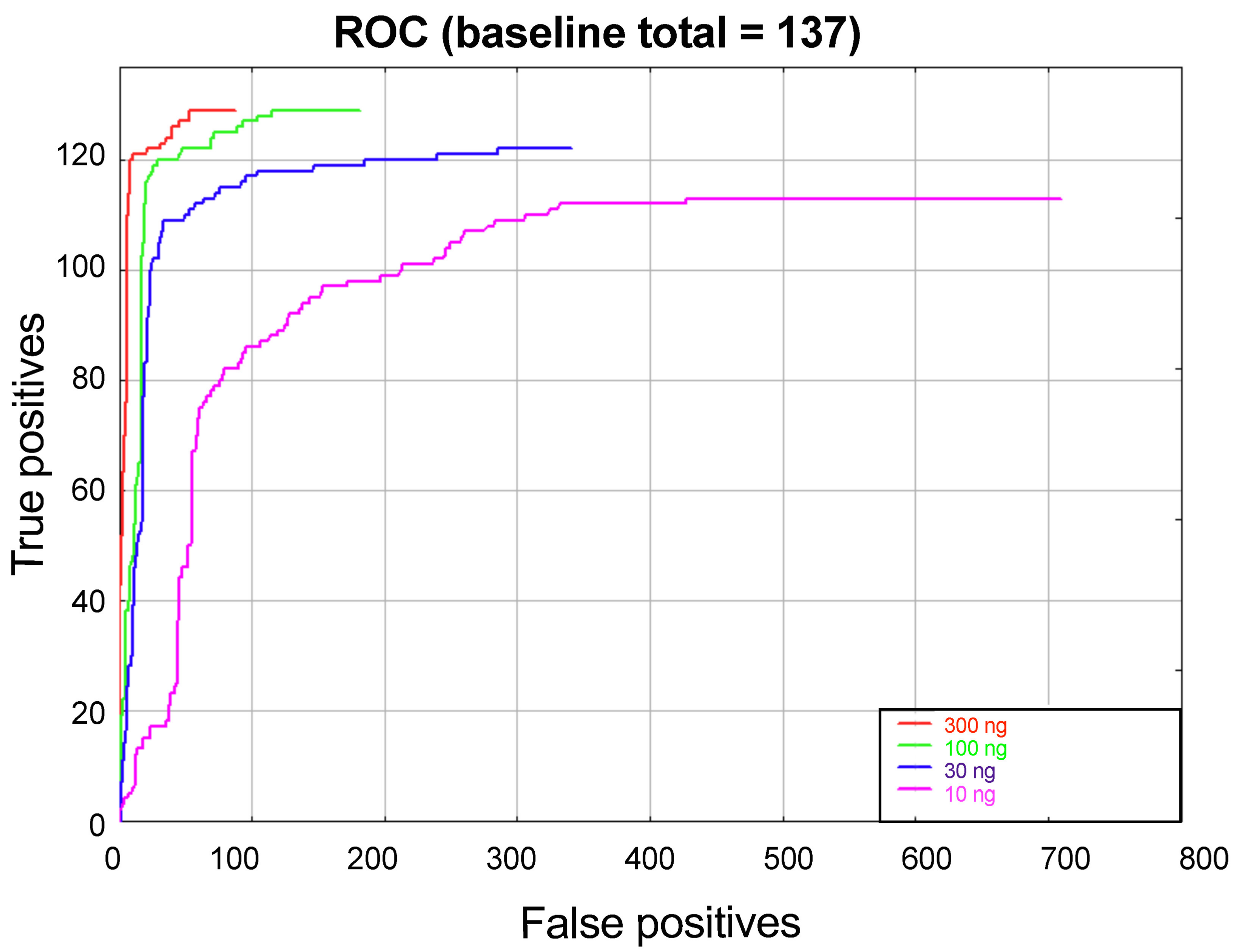

Supplementary Figure 3. Performance curve for variant detection.

A receiver-operator curve **(ROC)** showing true positives and false positives from one representative replicate of NA12878 at 300 (red line), 100 (green line), 30 (purple line), and 10 (pink line) ng input. The total variants included 128 single nucleotide variants (SNVs), and 9 insertions and deletions (indels).

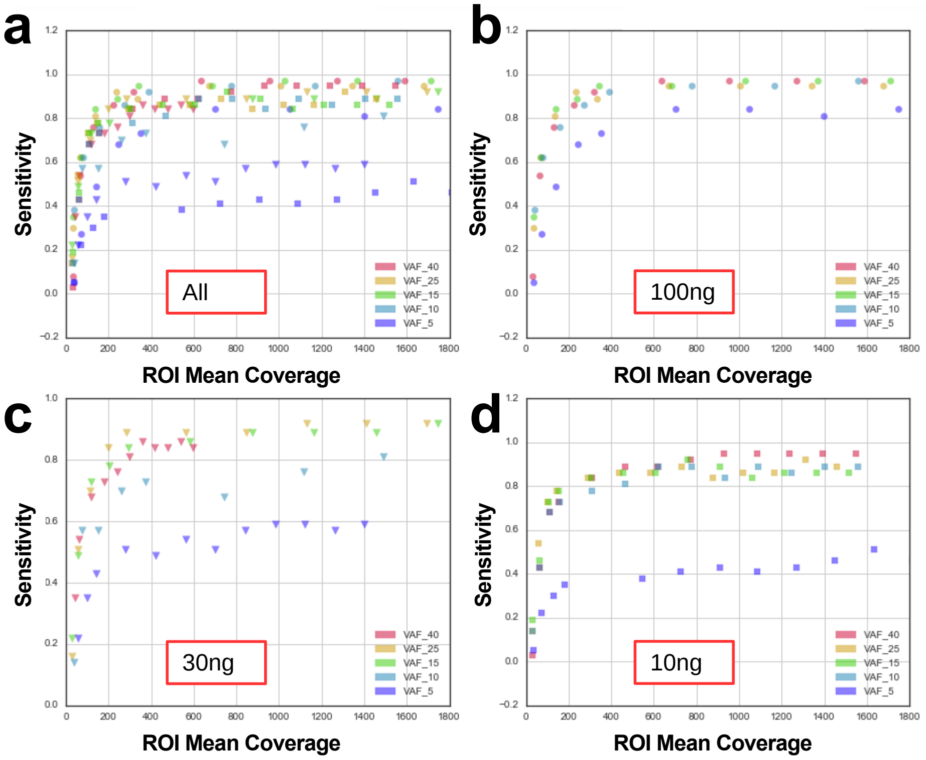

Supplementary Figure 4. Subsampling of the STMM-Mix-II SNV reference experiment.

To obtain a minimal threshold of mean coverage below which sensitivity is affected, we performed subsampling of the STMM-Mix-II SNV reference materials (Metods) and evaluated sensitivity at each subsampling threshold. We found that once mean coverage dropped below 200X, sensitivity was significantly affected. Pink: 40% VAF, orange: 25% VAF, Green: 15% VAF, light blue: 10% VAF, dark blue: 5% VAF, circles: 100 ng, triangles: 30 ng, squares: 10 ng. (a) depicts data from all input samples, (b) from 100 ng, (c) from 30 ng, and (d) from 10 ng.

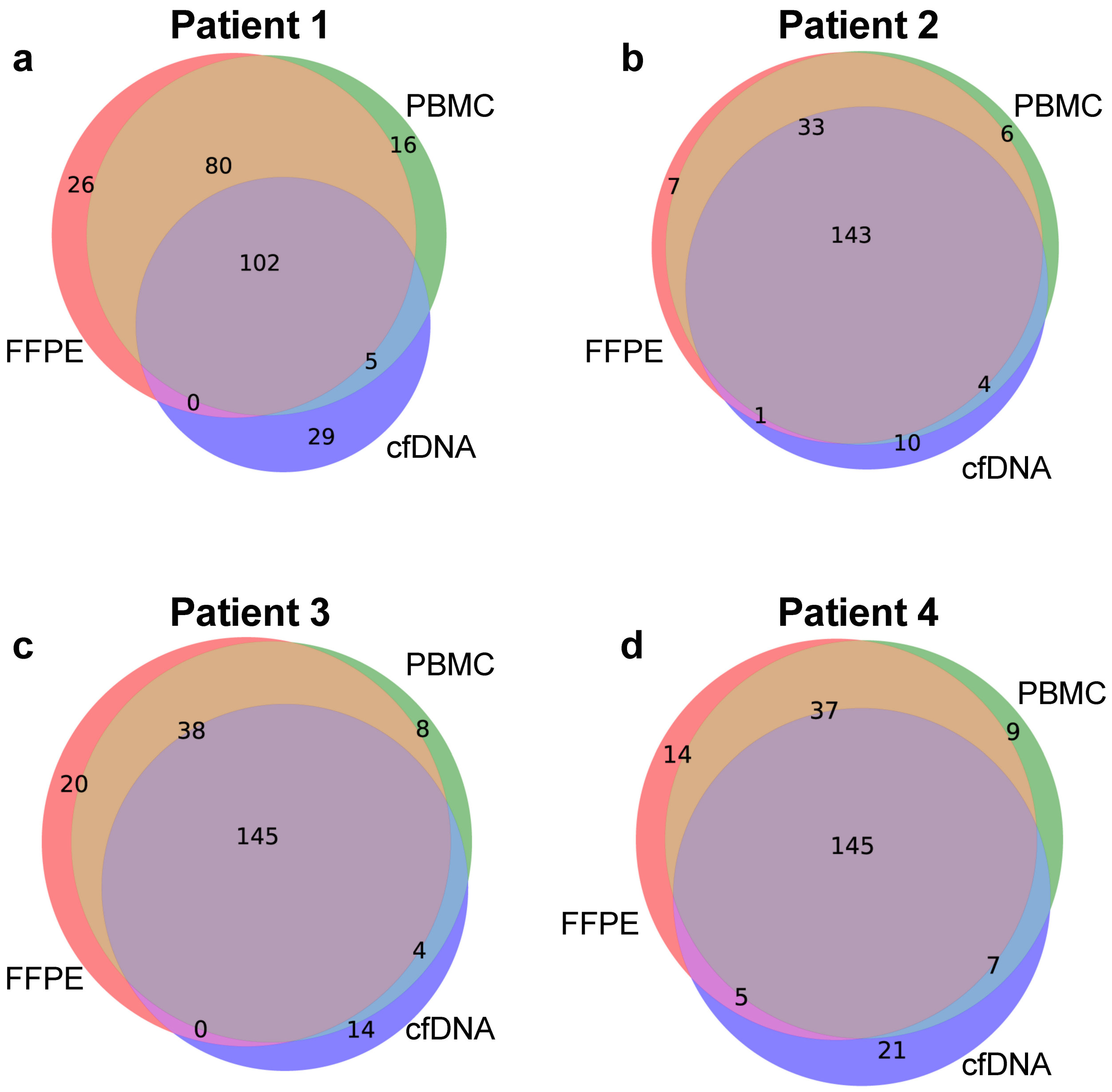

Supplementary Figure 5. Variant overlap from matched clinical samples.

Overlap of variants called by GATK in the FFPE (red circles), PBMC (green circles), and cfDNA (blue circles) samples from the four patients included in the matched sample study. (a-d) shows patient 1-4, respectively.

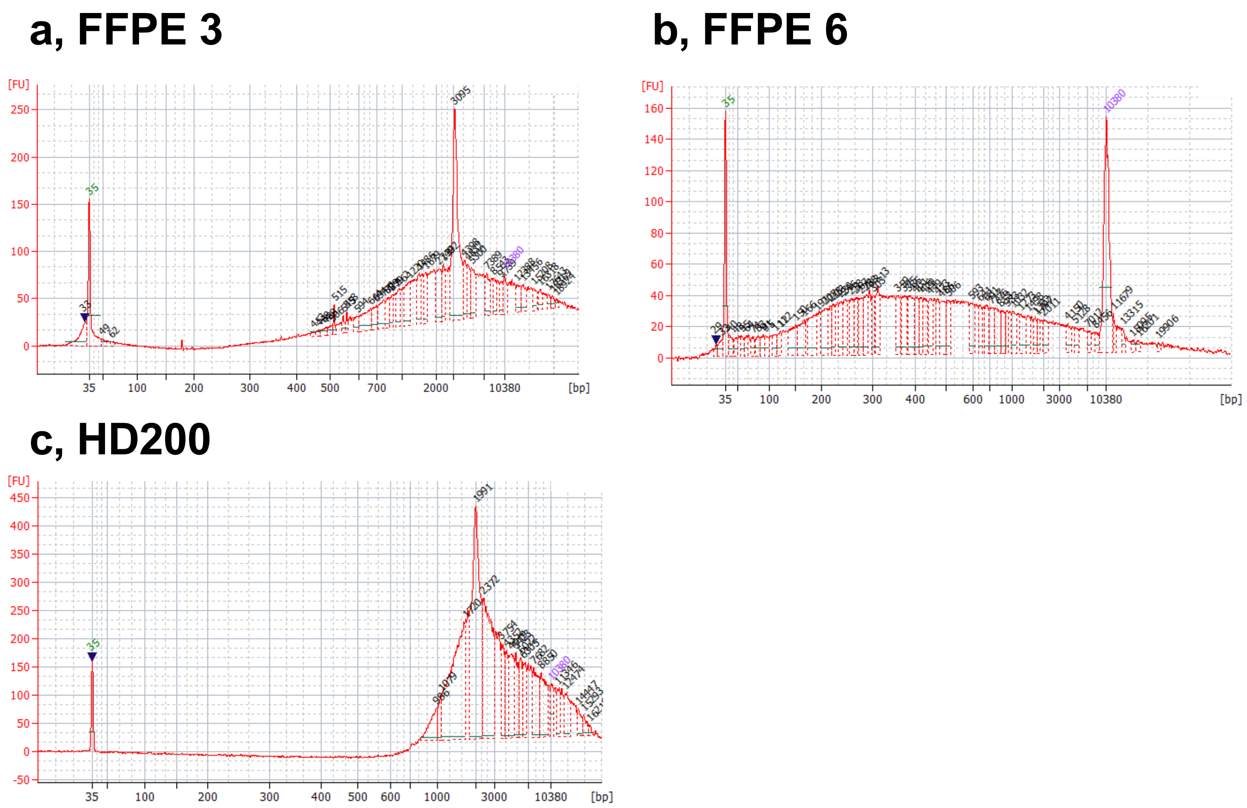

Supplementary Figure 6. DNA fragmentation in select FFPE derived DNA samples.

To investigate the level of DNA fragmentation in select FFPE samples at input (before sonication), two clinical FFPE samples (FFPE 3 (a) and 6 (b) as listed in Supplementary table 2) as well as the HD200 FFPE reference sample (c) were analyzed on the Bioanalyzer 2100. The figure shows that DNA fragmentation ranged from low in FFPE sample 3 and in HD200 to significant in FFPE sample 6 with an average fragment size of ~ 300 bp.

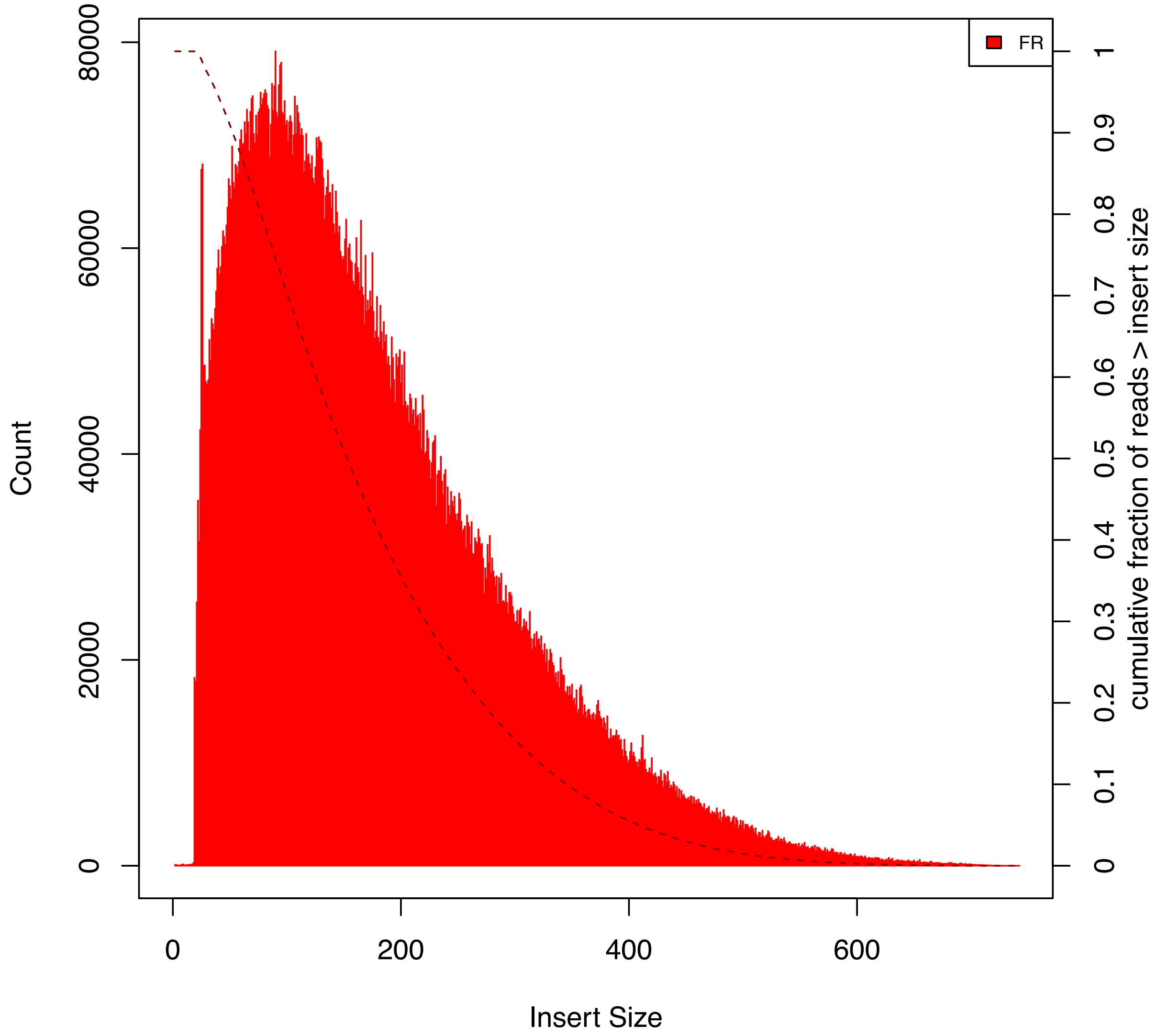

Supplementary Figure 7. Insert size measured by sequencing in NA12878.

The figure shows insert size measured by sequencing (detailed in the Methods section) for one sample of NA12878 at 100 ng. 3 additional replicates showed virtually identical patterns. Individual fragments shown in red and the dotted line shows the cumulative fraction of reads larger than insert size.

Supplementary Table 1. 130-gene list.

ABL1	*BRCA1*	*CDKN2B*	*FGFR1*	*KIT*	*NBN*	*PTPN11*	*SYK*
ABL2	*BRCA2*	*CEBPA*	*FGFR2*	*KRAS*	*NF1*	*RAD50*	*TET2*
AKT1	*BTK*	*CSF1R*	*FGFR3*	*MAP2K1*	*NFE2L2*	*RB1*	*TP53*
AKT3	*CALR*	*CTNNB1*	*FGFR4*	*MAP2K2*	*NOTCH1*	*RET*	*TPMT*
ALK	*CCND1*	*CYP2D6*	*FLT3*	*MAPK1*	*NPM1*	*RICTOR*	*TSC1*
APC	*CCND2*	*DDR2*	*GNA11*	*MCL1*	*NRAS*	*RNF43*	*TSC2*
AR	*CCND3*	*DNMT3A*	*GNAQ*	*MDM2*	*NTRK1*	*ROS1*	*U2AF1*
ARAF	*CD274*	*DPYD*	*GNAS*	*MET*	*NTRK2*	*RPTOR*	*UGT1A1*
ASXL1	*CDH1*	*EGFR*	*H3F3A*	*MLH1*	*NTRK3*	*RUNX1*	*VEGFA*
ATM	*CDK12*	*EMSY*	*HNF1A*	*MPL*	*PALB2*	*SF3B1*	*VHL*
ATR	*CDK2*	*ERBB2*	*HRAS*	*MRE11A*	*PARP1*	*SLCO1B1*	*WT1*
ATRX	*CDK4*	*ERBB3*	*IDH1*	*MSH2*	*PARP2*	*SMAD4*	
AURKA	*CDK5*	*ERBB4*	*IDH2*	*MSH6*	*PDGFRA*	*SMARCB1*	
AURKB	*CDK6*	*ERCC2*	*IGF1R*	*MTOR*	*PIK3CA*	*SMO*	
AXL	*CDK8*	*ESR1*	*JAK2*	*MUTYH*	*PMS2*	*SRC*	
BCL2	*CDK9*	*ETV6*	*JAK3*	*MYC*	*PTCH1*	*SRSF2*	
BRAF	*CDKN2A*	*EZH2*	*KDR*	*MYD88*	*PTEN*	*STK11*	

Supplementary Table 2. Sequencing metrics

Cell line, tissue sample or DNA	Input DNA (ng)	No. of total reads	% of total reads mapped	% on target bases	Mean target coverage	Fold 80 penalty	% of ROI bases 0x	% of ROI bases >2x	% of ROI bases >20x	% of ROI bases >100x
HD753	100	110205802	94%	55%	7310	1.86	0%	99%	99%	98%
		108749302	93%	57%	7423	1.83	0%	99%	99%	98%
		88135012	94%	57%	6089	1.86	0%	99%	99%	98%
NA12878 (CNV control)	100	75802056	94%	57%	5267	1.77	0%	99%	99%	98%
HD753	30	63178320	93%	56%	4263	1.91	0%	99%	98%	97%
		58532866	92%	54%	3797	1.93	0%	99%	98%	97%
		54430122	93%	56%	3701	1.94	1%	99%	98%	97%
NA12878 (CNV control)	30	33507542	93%	56%	2275	2.04	1%	99%	98%	96%
HD753	10	55559294	92%	53%	3575	2.30	1%	98%	97%	96%
		61116796	93%	50%	3678	2.32	0%	98%	97%	95%
		79969534	93%	51%	4882	2.13	1%	99%	98%	97%
NA12878 (CNV control)	10	40515504	93%	53%	2571	2.35	1%	98%	97%	95%
PBMC 1	100	35454871	92%	51%	2315	2.01	0%	99%	98%	96%
PBMC 2		60819476	93%	53%	4110	2.08	0%	99%	98%	97%
PBMC 3		83222324	93%	53%	5668	1.93	0%	99%	99%	98%
PBMC 4		48977047	93%	54%	3344	2.02	0%	99%	98%	97%
FFPE 1	100	20743673	94%	61%	1545	2.45	0%	98%	97%	95%
FFPE 2		56571084	94%	59%	4056	2.35	0%	99%	98%	97%
FFPE 3		48429980	94%	60%	3555	2.61	0%	99%	98%	96%
FFPE 4		46403043	94%	62%	3434	2.65	0%	99%	98%	96%
cfDNA 1	40	20795680	90%	43%	978	4.01	1%	96%	92%	87%
cfDNA 2		160425530	91%	44%	7780	2.19	1%	99%	98%	97%
cfDNA 3		72310096	91%	44%	3615	2.16	1%	99%	98%	96%
cfDNA 4		58667092	90%	44%	2853	2.48	1%	98%	97%	95%
NA12878	300	31367555	96%	85%	3272	1.78	0%	99%	99%	98%
		29441285	96%	85%	3063	1.75	0%	99%	99%	98%
		28539186	95%	85%	2975	1.76	0%	99%	99%	98%
		29487621	95%	85%	3080	1.77	0%	99%	99%	98%
	100	30501299	95%	78%	2917	1.96	0%	99%	98%	97%
		33446725	94%	79%	3205	1.97	0%	99%	98%	97%
		30097328	95%	79%	2894	1.98	0%	99%	98%	97%
		32297098	94%	79%	3097	1.95	0%	99%	98%	97%
	30	22911375	95%	78%	2257	2.23	1%	98%	97%	95%
		24544655	95%	79%	2453	2.17	1%	98%	97%	95%
		25614509	95%	77%	2499	2.15	0%	98%	97%	95%
		21908500	95%	78%	2160	2.23	0%	98%	97%	95%
	10	32151176	94%	67%	2710	3.49	1%	97%	94%	91%
		35590530	88%	70%	3149	3.15	1%	98%	96%	94%
		31205593	94%	63%	2497	3.89	1%	97%	94%	91%
		30386942	94%	68%	2585	3.76	1%	97%	95%	92%
HD200	300	32373402	95%	74%	3042	1.92	0%	99%	98%	97%
		40156181	95%	73%	3701	2.02	0%	99%	98%	97%
		56043828	95%	72%	5082	1.91	0%	99%	98%	97%
		66106561	95%	71%	5939	1.96	0%	99%	98%	97%
	100	70389501	93%	66%	5818	2.14	0%	99%	98%	97%
		57300213	94%	66%	4768	2.14	0%	99%	98%	97%
		52017269	95%	65%	4196	1.96	0%	99%	98%	97%
		40061295	94%	65%	3254	1.97	0%	99%	98%	96%
	30	30013711	94%	62%	2304	2.33	1%	98%	97%	94%
		35324044	95%	62%	2739	2.31	1%	98%	97%	95%
		46140323	95%	61%	3478	2.32	0%	98%	97%	95%
		33036732	95%	62%	2545	2.21	0%	98%	97%	95%
	10	24773897	95%	62%	1905	2.55	1%	97%	95%	93%
		24356873	95%	62%	1886	2.54	1%	97%	96%	93%
		21575961	95%	61%	1635	2.72	1%	97%	95%	92%
		19210875	95%	61%	1459	2.75	1%	97%	95%	92%
STMM-Mix-II, 25% VAF	100	49094806	89%	57%	1698	3.07	0%	99%	97%	93%
STMM-Mix-II, 15% VAF		50576606	89%	56%	1739	3.09	0%	99%	97%	93%
STMM-Mix-II, 10% VAF		57019613	89%	57%	1966	2.95	0%	99%	97%	94%
STMM-Mix-II, 5% VAF		52400018	88%	56%	1805	3.02	0%	99%	97%	93%
FFPE 5, TOMA repair	100	21019706	90%	84%	1966	2.55	2%	98%	97%	93%
FFPE 5, No repair		8150810	52%	48%	439	2.99	3%	96%	94%	84%
FFPE 5, NEB repair		17768438	91%	83%	1633	2.67	2%	98%	97%	92%
FFPE 6, TOMA repair		20034896	89%	83%	1808	2.95	2%	98%	96%	92%
FFPE 6, No repair		7210412	90%	85%	674	5.28	4%	96%	93%	82%
FFPE 6, NEB repair		36982426	86%	79%	3084	2.98	2%	98%	97%	94%
FFPE 7, TOMA repair		37195202	89%	82%	3446	2.69	2%	98%	97%	94%
FFPE 7, No repair		12665816	92%	85%	1255	3.02	2%	97%	96%	91%
FFPE 7, TOMA repair		23536628	89%	81%	2150	2.99	2%	98%	97%	92%
FFPE 2 rerun, TOMA repair		9105974	90%	88%	1037	3.89	3%	96%	94%	86%
FFPE 2 rerun, No repair		9153164	92%	89%	1068	4.87	4%	96%	93%	85%
FFPE 2 rerun, NEB repair		22069784	92%	87%	2543	3.95	3%	97%	95%	90%

Supplementary Table 3. Variants for STMM-Mix-II (coordinates given for GRC37)

Chr	Position	Reference base	Alternative base	Gene	Amino acid substitution
1	43815009	G	T	*MPL*	p.Trp515Leu
1	115256529	T	C	*NRAS*	p.Gln61Arg
2	209113113	G	A	*IDH1*	p.Arg132Cys
3	41266124	A	G	*CTNNB1*	p.Thr41Ala
3	178936091	G	A	*PIK3CA*	p.Glu545Lys
3	178952085	A	G	*PIK3CA*	p.His1047Arg
3	178952149	C	CA	*PIK3CA*	p.Ter1069MetfsTer4
4	1803568	C	G	*FGFR3*	p.Ser249Cys
4	55141049	CAGCCCA	CAGCCCG,A	*PDGFRA*	p.Ser566GlnfsTer6
4	55152093	A	T	*PDGFRA*	p.Asp842Val
4	55599321	A	T	*KIT*	p.Asp816Val
5	112175639	C	T	*APC*	p.Arg1450Ter
5	112175957	A	AA	*APC*	p.Thr1556AsnfsTer3
5	170837543	C	CTCTG	*NPM1*	p.Trp288CysfsTer12
7	55242465	GGAATTAAG..	G	*EGFR*	p.Glu693_Ala697delinsdel
7	55249012	C	CGGT	*EGFR*	p.Asp717_Asn718insGly
7	55249071	C	T	*EGFR*	p.Thr737Met
7	55259515	T	G	*EGFR*	p.Leu813Arg
7	140453136	A	T	*BRAF*	p.Val208Glu
9	5073770	G	T	*JAK2*	p.Val617Phe
9	80409488	T	G	*GNAQ*	p.Gln7Pro
10	43617416	T	C	*RET*	p.Met918Thr
10	89717716	A	AA	*PTEN*	p.Pro248ThrfsTer5
10	89717774	AA	A	*PTEN*	p.Lys267ArgfsTer9
11	108117846	TGT	T	*ATM*	p.Cys353SerfsTer5
12	25398284	C	T	*KRAS*	p.Gly12Asp
13	28592642	C	A	*FLT3*	p.Asp835Tyr
14	105246551	C	T	*AKT1*	p.Glu17Lys
17	7577120	C	T	*TP53*	p.Arg273His
17	7577538	C	T	*TP53*	p.Arg116Gln
17	7577557	AG	A	*TP53*	p.Cys242AlafsTer5
17	7578406	C	T	*TP53*	p.Arg175His
17	7579423	GG	G	*TP53*	p.Ser90ProfsTer33
17	37880981	A	AGCATACGT	*ERBB2*	p.Glu755_Ala756insAlaTyrValMet
18	48603092	G	GT	*SMAD4*	p.Ala55GlyfsTer19
19	3118942	A	T	*GNA11*	p.Gln58Leu
20	57484420	C	T	*GNAS*	p.Arg201Cys

Supplementary Table 4. Detection of variants from a control DNA mixture (HD200).

Variant	Base change (genomic)	% VAF spiked in	300 ng				100 ng				30 ng				10 ng			
			coverage		VAF		coverage		VAF		coverage		VAF		coverage		VAF	
			mean	SD	mean	SD	mean	SD	mean	SD	mean	SD	mean	SD	mean	SD	mean	SD
BRAF (V600E)	g.chr7:140453136A>T	10.5	10108	4415	11%	2%	8309	1632	11%	4%	6275	2755	8%	3%	2580	545	6%	4%
KIT (D816V)	g.chr4:55599321A>T	10	4559	1967	9%	2%	4123	1209	12%	3%	3068	733	8%	7%	1346	329	7%	10%
EGFR (ΔE746 - A750)	g.chr7:55242465_55242479delGGAATTAAGAGAA	2	3833	1303	1%	0%	4487	1022	1%	1%	2760	735	1%	0%	ND	ND	ND	ND
EGFR (L858R)	g.chr7:55259515T>G	3	9356	2546	2%	0%	8876	2505	2%	2%	4894	1050	6%	6%	3565	1009	2%	2%
EGFR (T790M)	g.chr7:55249071C>T	1	5203	1991	1%	1%	4941	565	1%	1%	2360	857	2%	3%	1764	267	2%	3%
EGFR (G719S)	g.chr7:55241707G>A	24.5	5682	1298	22%	4%	5722	1042	16%	2%	3051	775	25%	13%	1934	946	50%	12%
KRAS (G13D)	g.chr12:25398281C>T	15	4725	2300	12%	2%	6445	4321	10%	8%	3708	2216	9%	3%	2027	1197	16%	15%
KRAS (G12D)	g.chr12:25398285C>T	6	4814	2303	5%	2%	6652	4390	4%	3%	3758	2226	3%	2%	2105	1203	4%	7%
NRAS (Q61K)	g.chr1:115256530G>T	12.5	2940	795	11%	3%	2693	622	11%	4%	1938	1005	11%	6%	1629	966	30%	25%
PIK3CA (H1047R)	g.chr3:178952085A>G	17.5	4512	1329	17%	5%	6115	2760	14%	4%	3262	1075	14%	7%	2556	903	18%	1%
PIK3CA (E545K)	g.chr3:178936082G>A	9.5	4117	1003	9%	4%	5769	3297	7%	4%	2777	352	2%	2%	ND	ND	ND	ND
ALK (P1543S)	g.chr2:29416326G>A	33	2840	832	17%	2%	2931	912	15%	12%	836	261	16%	13%	820	361	44%	39%
ABL2 (P986fs)	g.chr1:179077445_179077445delG	8	3171	917	7%	7%	3408	910	3%	3%	2809	1932	6%	2%	2042	512	6%	2%
APC (R2714C)	g.chr5:112179431C>T	33	6311	3214	16%	8%	4906	2207	16%	7%	2713	765	20%	9%	1790	674	22%	23%
BRCA2 (A1689fs)	g.chr13:32913559_32913559delA	33	5510	2563	21%	7%	5270	1199	22%	6%	2496	777	21%	6%	1442	633	28%	23%
FGFR1 (P150L)	g.chr8:38285611G>A	8.5	4424	2341	5%	2%	3176	940	10%	6%	1730	825	11%	4%	1582	111	11%	16%
FLT3 (S985fs)	g.chr13:28578215_28578216delGA	10.5	3703	984	9%	3%	4565	771	8%	3%	2757	757	9%	6%	2198	1316	17%	5%
FLT3 (V197A)	g.chr13:28626706A>G	11.5	3590	957	8%	3%	5006	1387	7%	3%	2425	667	1%	2%	2124	982	23%	14%
IDH1 (S261L)	g.chr2:209106786G>A	10	4823	1182	9%	2%	4630	584	9%	2%	3125	361	10%	7%	1788	547	4%	5%
MET (V237fs)	g.chr7:116339848_116339848delT	6.5	7788	2678	5%	1%	7626	2321	2%	1%	4246	625	6%	3%	2585	750	7%	9%
MLH1 (L323M)	g.chr3:37061883C>A	8.5	6443	1888	8%	3%	7516	4199	6%	4%	3807	1458	10%	8%	2753	1029	9%	5%
NF1 (L626fs)	g.chr17:29552144_29552144delT	7.5	3036	971	8%	2%	3023	934	8%	6%	3046	913	4%	3%	1013	702	39%	41%
NOTCH1 (P668S)	g.chr9:139409754G>A	31.5	2413	1024	25%	8%	2946	985	22%	10%	1145	351	30%	13%	1400	551	27%	9%
PDGFRA (G426D)	g.chr4:55138600G>A	33.5	9958	3572	25%	1%	10656	1335	24%	3%	7898	1515	20%	2%	4737	957	26%	11%

In orange: Variant observed in 3 out of 4 replicates
In red: Variant observed in 2 out of 4 replicates
ND: Not detected, or detected in 1 out 4 replicates only

Supplementary Table 5. Detection of variants from a control DNA mixture (HD753).

Mutation	Base change (genomic)	% VAF spiked in	100 ng				30 ng				10 ng			
			coverage		VAF		coverage		VAF		coverage		VAF	
			mean	SD	mean	SD	mean	SD	mean	SD	mean	SD	mean	SD
GNA11 (Q209L)	g.chr19:3118942A>T	5.60	11070	1107	4.80%	2.00%	4979	1288	6.80%	2.10%	6087	4630	9.60%	11.30%
AKT (E17K)	**g.chr14:105246551A>T**	**5.00**	**3097**	**618**	**11.20%**	**6.60%**	**1025**	**430**	**1.20%**	**1.50%**	**1882**	**416**	**41.60%**	**53.40%**
PIK3CA (E545K)	g.chr3:178936082G>A	5.60	7920	1535	7.10%	0.80%	3514	1178	11.70%	6.40%	4446	1830	2.60%	4.40%
PIK3CA (H1047R)	**g.chr3:178952085A>G**	**16.70**	**8285**	**1173**	**18.80%**	**3.00%**	**5322**	**1641**	**17.50%**	**0.90%**	**4861**	**636**	**14.70%**	**12.60%**
EGFR (V769_D770insASV)	g.chr7:55249009insGCCAGCGTG	5.60	4266	95	0.30%	0.30%	3583	240	0.20%	0.00%	ND	ND	ND	ND
EGFR (ΔE746 - A750)	**g.chr7:55242465_55242479delGGAATTAAGAGAAGC**	**5.30**	**3951**	**505**	**3.40%**	**4.80%**	**ND**	**ND**	**ND**	**ND**	**1375**	**1056**	**2.00%**	**2.80%**
EGFR (G719S)	g.chr7:55241707G>A	5.30	9546	1819	5.30%	1.30%	5465	1637	14.90%	13.40%	3710	1064	11.00%	9.70%
KRAS (G13D)	**g.chr12:25398281C>T**	**5.60**	**10889**	**2829**	**1.50%**	**1.10%**	**5831**	**1081**	**5.50%**	**5.70%**	**4990**	**1303**	**1.90%**	**2.80%**
NOTCH1 (P668S)	g.chr9:139409754G>A	5.00	3298	949	9.30%	3.30%	3313	176	4.00%	3.90%	1099	573	0.60%	0.10%
MET (V237fs)	**g.chr7:116339848_116339848delT**	**2.50**	**16442**	**2773**	**2.00%**	**1.10%**	**8991**	**655**	**1.30%**	**1.10%**	**8190**	**3022**	**4.80%**	**6.90%**
FLT3 (S985fs)	g.chr13:28578215_28578216delGA	5.60	10640	7179	4.70%	7.60%	3664	444	4.60%	6.50%	2877	363	6.80%	3.00%
BRCA2 (A1689fs)	**g.chr13:32913559_32913559delA**	**5.60**	**5911**	**936**	**5.40%**	**1.60%**	**4065**	**1039**	**11.30%**	**10.80%**	**3491**	**829**	**8.10%**	**6.10%**
BRAF (V600E)	g.chr7:140453136A>T	18.20	13928	2086	17.40%	3.70%	7124	1016	16.30%	6.20%	8153	3541	22.30%	18.60%

In red: Variant observed in 2 out of 3 replicates

ND: Not detected, or detected in 1 out 3 replicates only

SD: standard deviation

Supplementary Table 6. Characteristics of clinical tissue source

**Patient number**	1	2	3	4
**Tumor type**	colorectal	non-small cell lung	non-small cell lung	non-small cell lung
**Patient gender**	male	male	male	male
**Disease stage**	III B	III B	III A	III A

Supplementary Table 7. Reported variants from analysis of clinical tissue samples

Patient	Sample	Number of SNVs	Number of SNVS listed in ExaC	Percentage of SNVS listed in ExaC	Number of SNVS listed in 1000 GP	Percentage of SNVS listed in 1000 GP
1	PBMC	203	196	97%	189	93%
	FFPE	208	188	90%	176	85%
	cfDNA	136	109	80%	105	77%
2	PBMC	186	180	97%	173	93%
	FFPE	184	175	95%	169	92%
	cfDNA	158	147	93%	146	92%
3	PBMC	195	186	95%	180	92%
	FFPE	203	186	92%	175	86%
	cfDNA	163	149	91%	143	88%
4	PBMC	198	193	97%	183	92%
	FFPE	201	193	96%	179	89%
	cfDNA	178	160	90%	147	83%

Supplementary Table 8. Somatic mutations from clinical tumor samples.

Sample	Gene	Chromosome	Position	Wildtype base	Mutation base	Mutation type	Wildtype amino acid	Substitution amino acid	CADD score	Tumors in COSMIC	COSMIC mutation ID
1	*FGFR3*	4	1807192	A	T	missense	LYS	MET	27	0	
	APC	5	112164616	C	T	stop_gained	ARG	TER	39	35	COSM18848
	EGFR	7	55229272	C	T	missense	ARG	TRP	24.4	0	
	NOTCH1	9	139399282	C	T	missense	GLY	SER	29.5	0	
	NOTCH1	9	139401880	C	T	missense	GLY	SER	35	0	
	NOTCH1	9	139402504	G	T	missense	THR	LYS	24.4	0	
	NOTCH1	9	139412721	C	T	missense	ALA	THR	26.7	0	
	RET	10	43600457	C	T	missense	ALA	VAL	23.2	0	
	HNF1A	12	121416876	C	T	missense	ALA	VAL	24.5	0	
	AKT1	14	105240286	C	T	missense	ARG	HIS	24.4	0	
	TSC2	16	2103430	C	A	missense	LEU	MET	26.4	0	
	TSC2	16	2124201	C	T	missense,splic	ARG	CYS	27.6	0	
	NF1	17	29585422	A	G	missense	ARG	GLY	25.8	0	
	ERBB2	17	37879573	C	T	missense,splic	PRO	SER	20.8	0	
	RPTOR	17	78897382	C	T	missense	PRO	LEU	28.3	0	
	STK11	19	1220701	C	T	missense	SER	LEU	35	0	
	MAP2K2	19	4101030	C	T	missense	ARG	HIS	29.7	0	
	U2AF1	21	44513336	C	T	missense	ARG	GLN	24.2	0	
	CYP2D6	22	42526694	G	A	missense	PRO	SER	24.9	8	COSM4999522
2	*CDKN2A*	9	21971159	C	T	missense	GLY	SER	33	2	COSM12746
	CDKN2B	9	22006235	C	A	missense	MET	ILE	32	0	
	CCND1	11	69466039	G	A	missense	VAL	MET	32	0	
	AKT1	14	105246424	C	T	splice_donor	na	na	22.9	0	
	AR	23	66765085	G	A	missense	VAL	MET	27	0	
3	*FGFR3*	4	1808964	C	T	missense	PRO	LEU	23.2	0	
	HRAS	11	532668	C	T	missense	GLY	SER	22.2	0	
	RPTOR	17	78796081	C	T	missense	ALA	VAL	34	1	COSM4070538
	RUNX1	21	36259397	C	T	splice,intron	na	na	22.3	0	
4	*FGFR3*	4	1803410	A	G	missense	THR	ALA	24.4	0	
	ABL1	9	133759889	C	T	missense	ARG	TRP	34	0	
	RUNX1	21	36164595	C	T	missense	ARG	HIS	34	1	COSM4854845
	RUNX1	21	36259241	C	A	missense	ASP	TYR	34	0	

Supplementary Table 9. C>T transition rate in FFPE samples treated with different repair steps

Patient	Repair	total number of variants	C>T/G>A	Percentage
2	TOMA repair	698	257	37%
	NO repair	855	438	51%
	NEB repair	674	249	37%
5	TOMA repair	1258	723	57%
	NO repair	1750	1241	71%
	NEB repair	1457	839	58%
6	TOMA repair	1139	613	54%
	NO repair	1575	1048	67%
	NEB repair	1024	525	51%
7	TOMA repair	1027	523	51%
	NO repair	1892	1341	71%
	NEB repair	1112	593	53%
